# Supplementary material for: Determinants of enrollment in community based health insurance among Households in Tach-Armachiho Woreda, North Gondar, Ethiopia, 2019
Source: PLoS One. 2020 Aug 31;15(8):e0236027. doi: 10.1371/journal.pone.0236027 (PMC7458327; doi:10.1371/journal.pone.0236027)
Supplement: Attachment — Submitted filename: PONE-D-20-00655_reviewer.docx [file pone.0236027.s001.docx]

**Comments to the authors:**

The study by MUULKEN GENETU CHANIE and GOJJAM ESHETIE EWUNETIE identified determinants of enrollment in CBHI (community-based health insurance) among households in Tach-Armachiho district. The structured questionnaire revealed that, there was no significant association between marital-status, family size, educational status, distance, religion, and chronic illness in the uptake of CBHI. They showed that uptake of the scheme was significantly depends on the sex of the household head. They also demonstrated that female headed households were more likely to enroll as compared to male-headed households. These findings are similar to others conducted in Ghana, Mali and Senegal. Dissimilar to this, studies conducted in Fogera Woreda, North West Ethiopia, Burkina Faso and Nigeria found that male-headed households were more likely to enroll as compared to female headed households. There were few studies which founds that no association exist between household head and uptake of the scheme. The study also demonstrated the link between age of the head of the households, and uptake of the CBHI scheme. The manuscript is well written and presented. All of the important points are discussed in diligent manner. Few questions that can be discussed are given below:

1. Why the authors have chosen the different numbers of CBHI and non CBHI cases. The non CBHI cases are double to the CBHI cases? How this difference can change the outcome of the study?
2. Are the biased male cases in both CBHI and non CBHI case could influence the outcome?
3. The enrolled cases could be more including both CBHI and non CBHI cases. Are the authors think that this could influence the outcome of the study?
