# Supplementary material for: Determinants of enrollment in community based health insurance among Households in Tach-Armachiho Woreda, North Gondar, Ethiopia, 2019
Source: PLoS One. 2020 Aug 31;15(8):e0236027. doi: 10.1371/journal.pone.0236027 (PMC7458327; doi:10.1371/journal.pone.0236027)
Supplement: Attachment — Submitted filename: 3. Respose to Reviewers.docx [file pone.0236027.s002.docx]

Rebuttal letter	Date: May 30/2020

**PONE-D-20-00655**

Determinants of enrollment in Community based health insurance among Households in Tach-Armachiho Woreda, North Gondar, Ethiopia, 2019

**Muluken Genetu Chanie**

**PLOS ONE**

Dear all,

We would like to thanks for these constructive, building and improving comments on this manuscript that improved the substance and contents of our manuscript. We considered each comments and clarify questions of editors and reviewers on the manuscript thoroughly. Our point-by-point responses for each comment and questions are described in detailed on the following pages. Further, the details of changes were shown by track changes in the supplementary document attached.

| **Latest New** Editor questions/comments**:-** Manuscript #: PONE-D-20-00655 | Responses |
| --- | --- |
| 1. Please ensure you refer to Table 3 in your text, as if accepted, production will need this reference to link the reader to the table | Thank you for comments. It was an editorial error and corrected as Table 2. |
| 2. Please provide the name of the ethics committee or Institutional Review Board that is imposing sharing restrictions on your de-identified minimal data set. | Thank you for comments and request for non-author contact.  Institutional Review Board of University of Gondar: [nigussuworku29@gmail.com](mailto:nigussuworku29@gmail.com) |
| **New** Editor questions/comments**:-** Manuscript #: PONE-D-20-00655 | Responses |
| 1, Please upload a copy of Figure 2 which you refer to in your text on page 9. Or if the figure is no longer to be included as part of the submission please remove all reference to it within the text. | Thank you for constructive comments. We revised and we have got no Figure 2 file. |
| 2. Please ensure that you refer to Table 2 in your text as, if accepted, production will need this reference to link the reader to the Table. | Thank you for constructive comments. Table 2 is found at page 10-11, and cited at page 10 in the manuscript. |
| 3. Please note that PLOS journals require authors to make all minimal data underlying the findings described in their manuscript fully available without restriction at the time of publication, with rare ethical or legal exceptions: https://journals.plos.org/plosone/s/data-availability.  PLOS ONE describes the minimal data set as that which is used to reach the conclusions drawn in the manuscript with related metadata and methods, and any additional data required to replicate the reported study findings in their entirety. This may include: - The values behind the means, standard deviations and other measures reported - The values used to build graphs - The points extracted from images for analysis  If there are no restrictions on the public sharing of your data, please upload your data either as a Supporting Information file or to a stable, public repository. PLOS holds a list of recommended repositories here: https://journals.plos.org/plosone/s/recommended-repositories | Thank you for constructive comments. We discussed on the dataset issues and agreed for providing for those who request the Authors on reasonable request. |

| **Editor questions/comments:-** Manuscript #: PONE-D-20-00655 | | **Response** |
| --- | --- | --- |
| 1. Please ensure that your manuscript meets PLOS ONE's style requirements, including those for file naming. The PLOS ONE style templates can be found at   <http://www.journals.plos.org/plosone/s/file?id=wjVg/PLOSOne_formatting_sample_main_body.pdf> and <http://www.journals.plos.org/plosone/s/file?id=ba62/PLOSOne_formatting_sample_title_authors_affiliations.pdf> | | Thanks editor for your constructive comments. We reviewed the PLOS ONE style requirements including file naming in the manuscript. |
| 2. Please include additional information regarding the survey or questionnaire used in the study and ensure that you have provided sufficient details that others could replicate the analyses. If you developed and/or translated a questionnaire as part of this study and it is not under a copyright more restrictive than CC-BY, please include a copy, in both the original language and English, as Supporting Information. | | Dear editor thanks for your constructive comments.  The survey tools were adopted from reviewing a couple of previous literatures. They were written in English language. They were paraphrased accordingly and referenced in the main document. No tool was developed by the authors. |
| 3. We note that you have reported significance probabilities of 0 in places. Since p=0 is not strictly possible, please correct this to a more appropriate limit, eg 'p<0.0001 | | Thanks again for your constructive comments. We reviewed and corrected in the main document as it was editorial error. |
| 4. We note that you have indicated that data from this study are available upon request. PLOS only allows data to be available upon request if there are legal or ethical restrictions on sharing data publicly. For information on unacceptable data access restrictions, please see <http://journals.plos.org/plosone/s/data-availability#loc-unacceptable-data-access-restrictions>.   In your revised cover letter, please address the following prompts:  a) If there are ethical or legal restrictions on sharing a de-identified data set, please explain them in detail (e.g., data contain potentially identifying or sensitive patient information) and who has imposed them (e.g., an ethics committee). Please also provide contact information for a data access committee, ethics committee, or other institutional body to which data requests may be sent.  b) If there are no restrictions, please upload the minimal anonymized data set necessary to replicate your study findings as either Supporting Information files or to a stable, public repository and provide us with the relevant URLs, DOIs, or accession numbers. Please see <http://www.bmj.com/content/340/bmj.c181.long> for guidelines on how to de-identify and prepare clinical data for publication. For a list of acceptable repositories, please see <http://journals.plos.org/plosone/s/data-availability#loc-recommended-repositories>.  We will update your Data Availability statement on your behalf to reflect the information you provide. | | Thanks editor for valuable comments. We addressed the comments in the cover letter accordingly. |
| 5. Thank you for stating the following in the Acknowledgments Section of your manuscript:   "Funding - University of Gondar"  We note that you have provided funding information that is not currently declared in your Funding Statement. However, funding information should not appear in the Acknowledgments section or other areas of your manuscript. We will only publish funding information present in the Funding Statement section of the online submission form.  Please remove any funding-related text from the manuscript and let us know how you would like to update your Funding Statement. Currently, your Funding Statement reads as follows: "no" | | Thanks editor for constructive comments. We removed the funding statement from the Acknowledgement section of the manuscript. The online submision form for funding will be filled as University of Gondar which covered the finance of this study. |
| 6. Your ethics statement must appear in the Methods section of your manuscript. If your ethics statement is written in any section besides the Methods, please move it to the Methods section and delete it from any other section. Please also ensure that your ethics statement is included in your manuscript, as the ethics section of your online submission will not be published alongside your manuscript. | | Thanks editor for constructive and interesting comment.  The ethics statement has moved to the methods section of the manuscript and deleted in any other section of the paper. |
| **Reviewer #1:** Manuscript #: PONE-D-20-00655 | | |
| The aim of the present study is to identify factors affecting of enrollment in community based health insurance. The topic is out-side the ambit of my subject expertise and an expert from the relevant field is required to do better justice to the article. | Thanks reviewer for your comment. | |
| **Reviewer #2:** Manuscript #: PONE-D-20-00655 | | |
| 1. Why the authors have chosen the different numbers of CBHI and non CBHI cases. The non CBHI cases are double to the CBHI cases? How this difference can change the outcome of the study? | Thanks reviewer for your comments. Even though the idea of CBHI accounts decades, its practice is low in the study area. The cases (CBHI enrolled households) are very low as to the efforts to enroll all households by the FMoH of Ethiopia; because of negative perceptions towards CBHI enrollment as evidenced from this study findings, and controls (Non CBHI enrolled Households) are high in number in the area, doubling the controls increase sample size of the study. This will increase the reliability of the study findings. | |
| 2. Are the biased male cases in both CBHI and non CBHI case could influence the outcome? | Thank you reviewer for the comment.  Yes. Because their perception towards CBHI enrollment would be different: those with positive will enroll and are cases whereas those with negative will resist enrollment. Supported by this study finding. Males are less likely to enroll in CBHI than Female headed households. | |
| 3. The enrolled cases could be more including both CBHI and non CBHI cases. Are the authors think that this could influence the outcome of the study? | Thank again for the comment.  Cases were selected from those households which enrolled in CBHI, whereas controls were selected from those households which were not enrolled in CBHI in the study area during the study period. Therefore the study included CBHI enrolled households as cases only. | |
